# The cell cycle of *Staphylococcus aureus*: An updated review

**DOI:** 10.1002/mbo3.1338

**Published:** 2022-12-09

**Authors:** Maria D. Barbuti, Ine S. Myrbråten, Danae Morales Angeles, Morten Kjos

**Affiliations:** ^1^ Faculty of Chemistry, Biotechnology and Food Science Norwegian University of Life Sciences (NMBU) Ås Norway

**Keywords:** cell division, cell morphology, cell wall synthesis, chromosome segregation, division site selection

## Abstract

As bacteria proliferate, DNA replication, chromosome segregation, cell wall synthesis, and cytokinesis occur concomitantly and need to be tightly regulated and coordinated. Although these cell cycle processes have been studied for decades, several mechanisms remain elusive, specifically in coccus‐shaped cells such as *Staphylococcus aureus*. In recent years, major progress has been made in our understanding of how staphylococci divide, including new, fundamental insights into the mechanisms of cell wall synthesis and division site selection. Furthermore, several novel proteins and mechanisms involved in the regulation of replication initiation or progression of the cell cycle have been identified and partially characterized. In this review, we will summarize our current understanding of the cell cycle processes in the spheroid model bacterium *S. aureus*, with a focus on recent advances in the understanding of how these processes are regulated.

## Graphical abstract

DNA replication, chromosome segregation, synthesis of the septal cell wall, and cytokinesis occur concomitantly in staphylococci. The processes are tightly coordinated temporally and spatially.

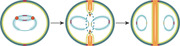

## 1 INTRODUCTION

Bacteria proliferate by consecutive rounds of cellular growth and division. During a cell cycle, the bacterial cell needs to replicate its DNA, segregate the new chromosomes, synthesize new cell walls and eventually divide. Proteins and protein complexes involved in these processes, have to exert their functions in a timely and spatially coordinated manner, to ensure that one cell, in the end, splits into two equal daughter cells, and the different processes need to be tightly controlled and regulated. Although cell cycle processes are, to a large extent, conserved across the bacterial kingdom, the exact mechanisms involved in this regulation differ, and unique species‐ or genus‐specific proteins and mechanisms play important roles. This also reflects the large diversity of cell morphologies and cellular lifestyles within the bacterial kingdom, including but not limited to cocci, bacilli, spirilla, vibrios, and spirochetes (Kysela et al., 2016; van Teeseling et al., 2017; Yang et al., 2016).

**Figure 1:**
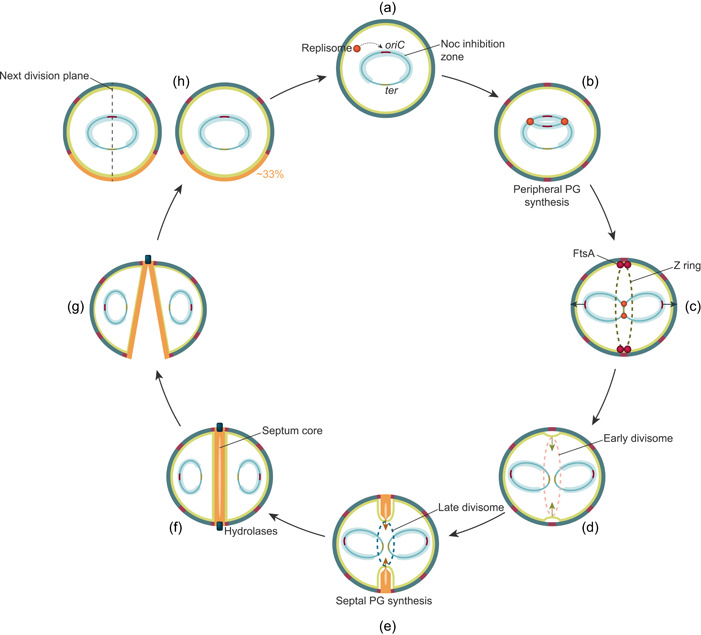
Schematic outline of the staphylococcal cell cycle. (a) Chromosome replication is initiated by binding of replisomes at the single origin of replication, *oriC*, after local DNA unwinding by DnaA. Noc‐bound DNA (light blue zone) prevents the assembly of FtsZ over the nucleoid. (b) The replisomes synthesize new DNA bi‐directionally from *oriC* and new PG is inserted along the cell periphery making the cell slightly elongated. (c) The two sister chromosomes are segregated to opposite sides of the cell, which results in a Noc‐free zone at midcell where FtsZ polymerizes to form a dynamic structure known as the Z ring. Proper Z ring assembly requires attachment to the cytoplasmic membrane via interactions with FtsA. (d) The Z ring acts as a scaffold for the recruitment of other conserved cell division proteins. The early divisome (important for regulating and stabilizing the Z ring) is recruited during the initial slow step of cytokinesis, which appears to be dependent on FtsZ treadmilling. During this step, initial invagination of the cytoplasmic membrane is observed. (e) MurJ is then recruited to the divisome, by the late divisome subcomplex DivIB‐DivIC‐FtsL, marking the turning point when cytokinesis becomes fast and likely dependent on PG synthesis and remodeling rather than FtsZ treadmilling. The late divisome synthesizes the septal cell wall (orange cell wall) and constricts the cytoplasmic membrane. The septum is thinner at the leading edge. (f) Peptidoglycan is inserted in the septum until it has attained a uniform thickness. The complete septum has separate layers with different PG architectures; an inner core of ordered PG with ring‐like architecture (light orange core) between two layers of mesh‐like PG, which is most likely synthesized by distinct PG synthesis enzymes. (g) The septum is presumably held together by the peripheral PG layer as it is synthesized. Hydrolases likely trigger cell splitting by degrading this bridge, not the entire septum, and this, together with mechanical factors, results in a sudden crack that separates the cells within milliseconds. (h) The daughter cells separate and the splitting of the septum generates approximately one‐third of the cell surface of each new cell. The next daughter cell division planes will be perpendicular to the previous ones.

The Gram‐positive pathogen *Staphylococcus aureus* is among the best‐studied coccus‐shaped bacteria. With the rise and spread of antibiotic resistance, including methicillin‐resistant and vancomycin‐resistant *S. aureus* (MRSA and VRSA, respectively), there is an urgent and continuous need to explore novel therapeutic targets in this priority pathogen (Tacconelli et al., 2017). Essential cell cycle processes, such as DNA replication and peptidoglycan synthesis, are well‐established antibiotic targets, but mechanisms critical for bacterial cell division also represent promising targets for novel antibiotics (Lock & Harry, 2008; Sass & Brötz‐Oesterhelt, 2013). To fully appreciate the repertoire of potential, but yet underexploited antibiotic targets in the bacterial cell cycle, it is critical to understand how the proteins involved work and how different processes are functionally linked. In this review, we summarize the current knowledge of cell cycle processes in *S. aureus*, with a focus on recent advances in the understanding of how these processes are regulated (Figure 1). We first discuss the mechanisms involved in chromosome replication and segregation, followed by an overview of the key features in staphylococcal cell wall biosynthesis. Then, the different stages of cell division are described, and finally, we discuss the mechanisms regulating and coordinating the different cell cycle processes, including recently identified factors that have been shown to modulate staphylococcal cell division and morphogenesis.

## 2 CHROMOSOME REPLICATION AND SEGREGATION

### 2.1 DNA replication

*S. aureus* possesses a single circular chromosome of approximately 2.8 Mb (Kuroda et al., 2001). Similar to other bacteria, replication of the chromosomal DNA is initiated by binding of the replication initiation factor DnaA to AT‐rich sequences (DnaA‐boxes) within the single origin of replication, *oriC* (Figure 1a). DNA is locally unwinded and the multiprotein replication machineries, known as the replisomes, are assembled into two replication forks (Briggs et al., 2012). New DNA strands are then synthesized bi‐directionally from *oriC* (Figure 1b) until the replication terminus, *ter* is reached and the replisomes dissolve (Hajduk et al., 2016; Katayama et al., 2010). Following replication termination, chromosome dimers are resolved by a dedicated recombinase system known as XerCD/*dif* (Midonet & Barre, 2014).

The bacterial replication machinery has mainly been studied in *Escherichia coli* and *Bacillus subtilis*, and consists, in addition to DNA polymerases, of proteins required for unwinding double‐stranded DNA, priming synthesis, and processivity. Replisome components and mechanisms are highly conserved in bacteria (see Beattie & Reyes‐Lamothe, 2015; Oakley, 2019 for reviews), nevertheless, there are also notable differences between the well‐studied bacterial model species. For example, Gram‐positive bacteria require two distinct polymerases for DNA replication (DnaE and PolC) in contrast to *E. coli*, which only use one polymerase. Furthermore, the spatial dynamics of the replisomes also appear to be variable between species (Bates, 2008) and it is debated whether replisomes are stationary replication factories or more mobile, independent complexes. Specific data on the staphylococcal replisome is only starting to emerge (Fagan et al., 2021), and it is not yet known whether any of these scenarios are suitable for describing the dynamics of the staphylococcal replisomes.

Some bacterial species, such as *E. coli* and *B. subtilis*, can perform multifork replication under fast‐growth conditions, meaning that they initiate a new DNA replication from *oriC* before the previous round has finished (Skarstad & Katayama, 2013). Wild‐type *S. aureus*, however, does not seem to have such multifork replication under normal growth conditions, as indicated by an *oriC*‐to‐*ter* ratio of approximately 2 during exponential growth (Gallay et al., 2021; Pang et al., 2017; Slager et al., 2014). One round of chromosome replication thus finishes before the next is initiated, and this occurs once per cell division cycle. Control of DNA replication initiation is a critical point of cell cycle regulation and multiple mechanisms are needed to coordinate DnaA activity with cell division (J. D. Wang & Levin, 2009). For example, overinitiation (i.e., *oriC*‐to‐ter ratio higher than 2) is observed in cells where DNA replication is disturbed by antibiotics or by mutations in genes controlling DnaA activity (Gallay et al., 2021; Pang et al., 2017; Slager et al., 2014). *noc* (nucleoid occlusion factor) is one of the genes with such a role. Noc is a known division inhibitor that controls the assembly of the division ring (see Section 5.2 for details) (Veiga et al., 2011), which was later shown to also negatively regulate DNA replication initiation in a DnaA‐dependent manner in *S. aureus* (Pang et al., 2017). Another protein important in coordinating DNA replication with cell division is the recently discovered cell cycle regulator CcrZ (see Section 5.2 for more details), which, in contrast to Noc, acts as a positive regulator of DnaA in *Firmicutes* (Gallay et al., 2021). Knockdown of this gene resulted in reduced replication initiation in *S. aureus* (Gallay et al., 2021). The exact mechanism by which CcrZ activates DnaA remains unknown, however, CcrZ is most probably a kinase, implying that CcrZ may modulate DnaA activity by phosphorylating an intermediate molecule or protein (Gallay et al., 2021; Wozniak et al., 2022). CcrZ, in turn, may also work in conjunction with the replication inhibitor YabA, identified in *B. subtilis* (Gallay et al., 2021; Noirot‐Gros et al., 2006; Wozniak et al., 2022). However, the staphylococcal YabA‐homolog has not been studied, and exactly how these proteins act together to control replication initiation still needs to be determined. Furthermore, additional mechanisms are also probably involved. For example, the nucleoid‐associated protein HU was recently shown to directly affect the initiation of DNA replication in *B. subtilis* (Karaboja & Wang, 2022), and future studies will unravel whether HU has the same function in *S. aureus*.

### 2.2 Chromosome segregation

Following replication, the two sister chromosomes are segregated into two sister cell compartments (Figure 1c). While the details of this process remain elusive, some mechanisms are known to be important for proper chromosome segregation in *S. aureus*. These include the ParB/*parS*‐system (ParB is also known as Spo0J) and the structural maintenance of chromosomes (SMC) complex (H. Chan et al., 2020; Yu et al., 2010). ParB is a DNA‐binding protein that binds to specific *parS* sequences in the *oriC*‐proximal region (H. Chan et al., 2020; Gruber & Errington, 2009). The SMC protein is a condensin that, in complex with ScpA and ScpB, has an important role in the condensation and organization of the chromosome (Britton et al., 1998; Mascarenhas et al., 2002). Similar to the mechanisms in *B. subtilis* and *Streptococcus pneumoniae* (Minnen et al., 2011; Sullivan et al., 2009), H. Chan et al. (2020) have demonstrated that ParB and SMC co‐localizes and work together to maintain proper chromosome segregation in *S. aureus*. It was shown that correct localization of SMC is dependent on ParB, and deletion of *parB* and *smc* simultaneously increased the number of cells with chromosome segregation defects, although the viability of *S. aureus* was not severely affected. Most likely ParB is important for loading SMC onto the chromosomal origin, while the recombinase XerD unloads SMC from the chromosomes at the terminus (Karaboja et al., 2021). It should be noted that ParB, which is a Noc‐homolog, is known to affect DNA replication in *B. subtilis*, but ParB does not seem to have the same role in *S. aureus* (Pang et al., 2017).

Furthermore, DNA translocases coordinate chromosome segregation with septum closure by clearing the midcell of chromosomal DNA by actively pumping it across the division septum (see H. Chan et al., 2022 for a recent review). *S. aureus* is known to encode two putative DNA translocases, SpoIIIE and FtsK, and it has been shown that the cells require one of these proteins for normal chromosome segregation (Veiga & Pinho, 2017). By examining cells with almost completed septa, Veiga and Pinho (2017) observed that SpoIIIE concentrated in foci inside the septum opening in ∼50% of the cells, where SpoIIIE is thought to actively pump DNA away from being bisected by the septum (Table 1).

**Table 1:** Overview of different cell cycle factors discussed in this review

Gene name	Locus tag^a^ SAOUHSC_	Locus tag^b^ USA300_	Involvement in the cell cycle
DnaA	_00001	_0001	DNA replication, replication initiation
XerC	_01224	_1145	DNA replication, recombinase
XerD	_01591	_1447	DNA replication, recombinase
Noc	_00342	_0361	DNA replication initiation and cell division control protein
YabA	_00456	_0463	Putative DNA replication initiation control protein^c^
CcrZ	_01866	_1695	DNA replication initiation and cell division control protein
HU	_01490	_1362	DNA binding protein, putative DNA replication initiation control^c^
DnaD	_01470	_1344	Primosomal protein, putative DNA replication initiation control^c^
ParB/Spo0J	_03049	_2643	Chromosome organization and segregation
SMC	_01204	_1127	Chromosome organization and segregation
SpoIIIE	_01253	_1169	Chromosome segregation, DNA translocase
FtsK	_01857	_1687	Chromosome segregation, DNA translocase
MurJ	_01871	_1700	PG synthesis, lipid II flippase
PBP1	_01145	_1075	PG synthesis, transpeptidase works in conjunction with FtsW
PBP2	_01467	_1341	PG synthesis, bifunctional transpeptidase and transglycosylase
PBP3	_01652	_1512	PG synthesis, transpeptidase works in conjunction with RodA
PPB4	_00646	_0629	PG synthesis, transpeptidase and carboxypeptidase
PBP2a	n.p.	_0032	PG synthesis, present in MRSA strains
FtsW	_01063	_1013	PG synthesis, transglycosylase, works in conjunction with PBP1
RodA	_02319	_2040	PG synthesis, transglycosylase, works in conjunction with PBP3
SgtA	_01840	_1676	Transglycosylase
SgtB	_02012	_1855	Transglycosylase
FtsZ	_01150	_1080	Cell division, major early cell division protein
FtsA	_01149	_1079	Cell division, early, FtsZ‐interaction
SepF/YlmF	_01154	_1083	Putative early cell division, FtsZ‐interaction^c^
EzrA	_01164	_1664	Cell division, early, FtsZ‐interaction and regulation
ZapA	_01096	_1040	Putative early cell division protein interacting with FtsZ^c^
GpsB	_01462	_1337	Cell division, late, Z ring stabilization
DivIB/FtsQ	_01148	_1078	Cell division, late
DivIC/FtsB	_00482	_0485	Cell division, late
FtsL	_01144	_1074	Cell division, late
Stk1/PknB	_01187	_1113	Serine/threonine protein kinase
Stp1	_01186	_1112	Serine/threonine protein phosphatase
CozEa	_00948	_0193	Cell division and morphology determinant
CozEb	_01358	_1254	Cell division and morphology determinant
SmdA	_01908	_1729	Morphology determinant
SosA	_01334	n.a.	Cell division inhibitor
DivIVA	_01158	_1086	No known function in cell division^d^
MreC	_01759	_1605	No known function in cell division^d^
MreD	_01758	_1604	No known function in cell division^d^

Abbreviations: n.a., not annotated; n.p., not present.

^a^ Locus tag in model strain NCTC8325.

^b^ Locus tag in model strain USA300 JE2.

^c^ The involvement of these proteins in cell division is predicted based on studies from other bacteria, but has not been studied specifically in *S. aureus*.

^d^ Proteins associated with the cell cycle in other bacteria, but published studies suggest that they have no such role in *S. aureus*.

Neither SMC/ParB nor SpoIIIE/FtsK is essential, although combined deletions increase chromosome management defects (H. Chan et al., 2020; Veiga & Pinho, 2017). Therefore, it seems likely that *S. aureus* has several systems, partially overlapping, to ensure efficient chromosome segregation. Furthermore, the lack of essentiality of these proteins suggests the involvement of additional passive processes in chromosomal segregation, such as DNA replication, DNA transcription, and entropic forces, as concluded from studies in other bacteria and by computer modeling (Dworkin & Losick, 2002; Gogou et al., 2021; Jun & Wright, 2010; Kjos & Veening, 2014; Lemon & Grossman, 2001; Pinho et al., 2013; Saraiva et al., 2020). Physical models proposing that chromosome segregation may result largely from entropic forces argue that chromosomes will repel each other to maximize their total conformational entropy under strongly confining conditions, like in the cytoplasm (Jun & Wright, 2010). Indeed, the staphylococcal chromosome occupies nearly the entire cytoplasmic space fitting the physical conditions described for the entropy‐driven segregation mechanism, and physical confinement and spatial constraints have been suggested to determine the orientation of chromosome segregation in *S. aureus*, occurring in parallel with the septum (Figure 1c) (Saraiva et al., 2020). However, the importance of such passive processes varies between bacterial species (Dworkin & Losick, 2002; Kjos & Veening, 2014; Lemon & Grossman, 2001; Migocki et al., 2004; Reyes‐Lamothe et al., 2008; X. Wang & Sherratt, 2010) and further experimental data on *S. aureus* is needed.

## 3 THE STAPHYLOCOCCAL CELL WALL

The staphylococcal cell wall, which is approximately 20 nm wide (Pasquina‐Lemonche et al., 2020), consists of a mesh of peptidoglycan (PG) and teichoic acids which encloses the cytoplasmic membrane. Collectively, the PG and teichoic acids provide structural support against the high internal turgor pressure of the cell (up to 20 atm in Gram‐positive bacteria [Whatmore & Reed, 1990]) to maintain the cell shape throughout the cell cycle.

### 3.1 Peptidoglycan structure and biosynthesis

**Figure 2:**
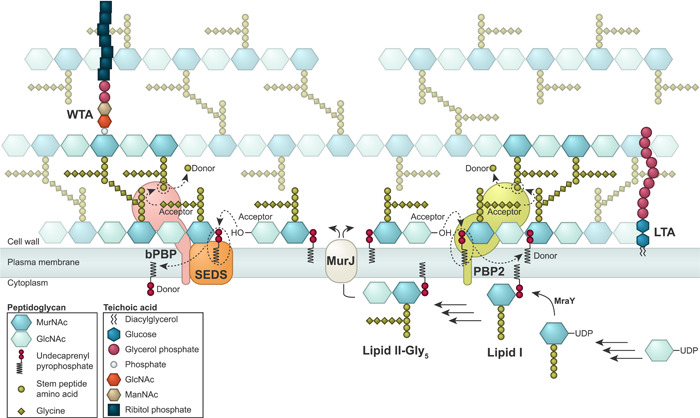
Overview of cell wall biosynthesis in *Staphylococcus aureus*. The core unit of PG, lipid II‐Gly_5_, is a β‐1 → 4 linked N‐acetylglucosamine (GlcNAc)‐N‐acetylmuramic acid (MurNAc) disaccharide with a stem peptide (consisting of L‐alanine, D‐iso‐glutamine, L‐lysine, and two D‐alanine residues) attached to MurNAc with a pentaglycine attached to the L‐lysine. This molecule is synthesized in the cytoplasm, where the precursor fructose‐6‐phosphate is converted to the uridine diphosphate (UDP)‐linked sugar precursors UDP‐N‐acetylglucosamine (UDP‐GlcNAc) and UDP‐MurNAc in a series of reactions. The stem peptide amino acids are attached consecutively, to make the UDP‐MurNAc‐pentapeptide. Next, the membrane protein MraY catalyzes the linking of the MurNAc‐pentapeptide to a lipid undecaprenyl phosphate carrier (C_55_‐P, also known as bactoprenol) anchored on the cytoplasmic side of the membrane, making lipid I. Lipid II is then formed by MurG, which adds GlcNAc derived from UDP‐GlcNAc to lipid I. Still, at the inner surface of the plasma membrane, lipid II is modified by the stepwise addition of five glycines (Gly) residues to the third amino acid in the stem peptide, catalyzed by FemABX, and amidation of the D‐iso‐glutamic acid to D‐iso‐glutamine in position 2 of the stem peptide by the complex MurT‐GatD (not shown). At this point, Lipid II‐Gly_5_ is flipped to the outer side of the cytoplasmic membrane by MurJ. The PG mesh is composed of glycan chains with an average length of 3‐10 disaccharides which are linked by pentaglycine cross‐bridges between the L‐lysine of one stem peptide and the fourth D‐alanine of another. The elongation of glycan chains is catalyzed by transglycosylases (PBP2 or SEDS proteins FtsW and RodA) which add new Lipid II monomers (the acceptor) to the reducing end of the growing glycan chains (the donor). Crosslinking of the chains is catalyzed in transpeptidation reactions catalyzed by bPBPs who crosslink the fifth glycine residue of one peptide (the acceptor) to the fourth D‐alanine of another (the donor) with the release of the terminal D‐alanine of the donor peptide. Teichoic acids (LTA and WTA) are also important constituents of the staphylococcal cell wall. These are synthesized by separate machinery not indicated in the figure.

PG consists of glycan chains of various lengths that are linked via peptide bridges (Figure 2). The mature staphylococcal PG can be described as a porous, mesh‐like hydrogel (Pasquina‐Lemonche et al., 2020). To maintain cell morphology and integrity during cell growth and division, PG synthetases and hydrolases work together to incorporate new PG into the existing mesh and to make the septal cross wall. Interestingly, using atomic force microscopy, it has been shown that the septum of staphylococci consists of two different peptidoglycan layers with distinct architectures; an inner, primary layer with highly ordered, ring‐like PG‐structures and outer layers with randomly oriented mesh, similar to the peripheral cell wall (Figure 1f) (Pasquina‐Lemonche et al., 2020; Su et al., 2020).

The biosynthesis pathway of PG (Figure 2) is highly conserved in bacteria and has been reviewed extensively elsewhere (Egan et al., 2020; Vollmer & Seligman, 2010). Briefly, PG biosynthesis initiates in the cytoplasm where (UDP)‐linked sugar precursors are converted to the membrane‐attached PG units known as lipid II, which is further modified to lipid II‐Gly_5_, by the addition of five glycines (Gly) residues to the third amino acid in the stem peptide (Figure 2) (Rohrer & Berger‐Bächi, 2003). These glycines are the basis of the unique pentaglycine bridges found in the highly crosslinked staphylococcal PG (Figure 2). Lipid II‐Gly_5_ is then flipped to the outer side of the cytoplasmic membrane by MurJ (Sham et al., 2014), which is the protein responsible for directing peptidoglycan synthesis to midcell for initiation of septum synthesis (Section 4.2) (Monteiro et al., 2018). In the final stage of PG synthesis, transglycosylation (TG, polymerization of subunits from lipid II to form the glycan chains) and transpeptidation (TP, crosslinking of stem peptides with pentaglycine bridges) take place at the surface of the cytoplasmic membrane. Penicillin‐binding proteins (PBPs) are the main proteins involved in this final stage together with FtsW and RodA, the latter two belonging to the family of shape, elongation, division, and sporulation (SEDS) proteins. In contrast to other well‐studied bacterial species, like *B. subtilis*, *E. coli*, and *S. pneumoniae* that contain between 6 and 16 PBPs each and have functionally distinct machinery for lateral and peripheral PG synthesis (Sauvage et al., 2008), *S. aureus* has been recognized as a simple model organism because it contains only four or five PBPs and mainly synthesize PG in one machinery at the septum (Pinho & Errington, 2003; Reed et al., 2015).

The staphylococcal PBPs include the bifunctional (class A PBPs, aPBP) PBP2 with both TGase and TPase activity, the two monofunctional (class B PBPs, bPBP) TPases PBP1 and PBP3, and PBP4 which is a low‐molecular‐mass PBP with TPase activity. In addition, MRSA strains encode a fifth PBP, the monofunctional TPase PBP2A (Hartman & Tomasz, 1984). Among these, only PBP1 and PBP2 are essential for growth. While all staphylococcal PBPs can cross‐link glycan strands (TPase activity), PBP2 was for a long time the only staphylococcal protein identified with TGase activity. However, a study from 2001 showed that the inactivation of the TGase domain of PBP2 did not abolish the elongation of nascent PG chains, suggesting that other transglycosylases play a role in *S. aureus* (Pinho et al., 2001). Two putative monofunctional glycosyltransferases designated SgtA and SgtB were identified more than 20 years ago by whole‐genome sequencing (Kuroda et al., 2001). They were later both reported to have glycosyltransferase activity in vivo, although only SgtB can support the growth of *S. aureus* in the absence of the TGase activity of PBP2 (Reed et al., 2011; Q. M. Wang et al., 2001). In recent years, a more complete understanding of this process emerged with the discovery of the SEDS proteins as a new family of PG polymerases harboring TGase activity (Meeske et al., 2016). The SEDS proteins RodA and FtsW in rod‐shaped *B. subtilis* were shown to work together with bPBPs in cognate TG–TP pairs, polymerizing lateral and septal PG, respectively. The presence of both RodA and FtsW in *S. aureus* thus raised the question of their role in cocci, perceived to only contain one division machinery due to, among others, their lack of MreB, the cytoskeletal protein responsible for elongation in rod‐shaped bacteria (Pinho et al., 2013). However, super‐resolution microscopy revealed that *S. aureus* is not fully spherical throughout the cell cycle, it does have some lateral PG synthesis before septum synthesis (Monteiro et al., 2015). Specifically, FtsW works in pair with PBP1 and RodA works in pair with PBP3 to mediate septal and lateral PG incorporation, respectively (Reichmann et al., 2019). The coordinated activity of these complexes is responsible for *S. aureus*' spheroid cell morphology. Consistent with their different functions, PBP1‐FtsW is essential for the viability of *S. aureus*, while PBP3‐RodA is not. Furthermore, although PBP1 is essential, mutants lacking PBP1 TPase activity are still viable, implying that this protein has other functions in addition to being the primary TPase. These functions are still elusive, however, PBP1 indeed plays a role in septum formation and progression independent of its TPase activity (Wacnik et al., 2022). It could therefore possibly act as a stabilizer of the FtsW‐PBP1 complex in the divisome at midcell (Reichmann et al., 2019) and/or stimulate the essential TGase activity of FtsW (Taguchi et al., 2019).

PBP2, the only bifunctional aPBP in *S. aureus*, has a septum‐enriched localization similar to the other PBPs (Monteiro et al., 2018; Pinho & Errington, 2005). Despite being essential, its exact function is not known. It has been suggested that while the SEDS‐bPBP pairs synthesize the primary PG in the septum, the bifunctional PBP2, may be involved in the synthesis of the mesh‐like PG layers (Section 4.2) and/or repair and maintenance of the PG mesh (Straume et al., 2021; Wacnik et al., 2022).

PBP4 functions as a secondary transpeptidase in *S. aureus*, responsible for the high degree of cross‐linking found in *S. aureus* PG (Atilano et al., 2010; Wyke et al., 1981). PBP4, whose cellular localization is known to be influenced by teichoic acids (Atilano et al., 2010), is additionally proven to be an important contributor to β‐lactam resistance (da Costa et al., 2018; Hamilton et al., 2017; Henze & Berger‐Bächi, 1995).

As mentioned above, MRSA strains encode a fifth PBP, the TPase PBP2a, which has been acquired by horizontal gene transfer and is responsible for their β‐lactam resistant phenotype. PBP2a (see Fishovitz et al., 2014 for review) has, in contrast to PBP1‐4, low affinity for most β‐lactams, and MRSA strains are thus able to perform crosslinking even in the presence of such antibiotics. PBP2a can replace the TPase function of the otherwise essential PBP1 and PBP2 in staphylococcal cells, but the TGase activity of PBP2 remains essential (Pereira et al., 2007; Pinho et al., 2001; Wacnik et al., 2022). The important role of this exogenous PBP suggests that the different PBPs fulfill different roles in MRSA and MSSA strains and underlines the importance of studying aspects related to cell wall synthesis in both types of strains.

### 3.2 PG hydrolases are critical for building the cell wall

In addition to the PG synthetases, PG hydrolases are also critical for building the new cell wall. The best‐characterized hydrolases, Atl and Sle1, are primarily known for their role in daughter cell splitting (Section 4.3), however, these enzymes are also important for cell wall growth and recycling. Their hydrolytic activity is also responsible for the porous mesh‐like architecture of the cell wall. PG hydrolases have different enzymatic activities; glucosaminidases and muramidases cut bonds within the glycan chain, amidases cut the bond connecting the stem peptide to the glycan chain, endopeptidases cut within the stem peptide, and carboxypeptidases cut off the terminal amino acid of the stem peptide (Vollmer et al., 2008). *S. aureus* encodes a large array of different PG hydrolases (see M. Wang et al., 2022 for review). Functional data on specific hydrolases have emerged in recent years. For example, the glucosaminidases SagA, SagB, Atl, and ScaH are involved in cell expansion after cell splitting (Wheeler et al., 2015) and the membrane‐bound amidase LytH is important for the early stage of cell division where it removes stem peptides from uncrosslinked PG and thereby controls PG synthase activity and cell expansion (Do et al., 2020).

To balance the activity of hydrolytic enzymes on PG during growth, while at the same time avoiding uncontrolled cell lysis, the activity of the hydrolases must be properly regulated. The regulation occurs at different levels, from transcriptional regulation, protein–protein interactions, proteolysis (Jensen et al., 2019; Kirsch et al., 2021), and localization control via interactions with teichoic acids (see Sections 3.3 and 5.3). Most of the hydrolases are transcriptionally regulated by one or several two‐component system(s), including the essential WalK/WalR (previously called YycG/YycF) (Dubrac & Msadek, 2004). The signal(s) sensed by the sensor kinase WalK is not known, but recent evidence from *B. subtilis* suggests this may be cleaved products from different hydrolases, that are used as a proxy for the maturity of the cell wall (Dobihal et al., 2019). Furthermore, direct regulators of hydrolase activity through protein–protein interactions have been identified recently, including ActH which forms a complex and activates LytH (Do et al., 2020) and the multitransmembrane spanning protein SpdC that complexes with SagB (Schaefer et al., 2021). The latter protein, SpdC, belongs to a large family of proteins with similarity to the eukaryotic CAAX proteases and it will be interesting to find out whether other proteins in this family play similar roles in controlling cell wall homeostasis.

### 3.3 Teichoic acids are important for cell shape

Teichoic acids are anionic glycopolymers covalently linked to either the PG (wall teichoic acid, WTA) or the membrane (lipoteichoic acid, LTA) (Figure 2). These polymers have a profound impact on the cell cycle progression and cell shape of *S. aureus*. The staphylococcal WTA consists of 11–40 unit chains of ribitol‐phosphate (RboP) (Neuhaus & Baddiley, 2003; Swoboda et al., 2010; Xia et al., 2010) that are linked to MurNAc in the PG. WTA polymers are synthesized in the cytoplasm by a number of enzymes (encoded by the *tar* genes) and then exported across the membrane by the ABC transporter TarGH (see Brown et al., 2013 for an overview of the staphylococcal WTA biosynthesis). The LytR‐CpsA‐Psr (LCP) proteins MsrR (LcpA) and SA0908 (LcpB) are suggested to be responsible for the anchoring of poly‐RboP to the PG (Y. G. Y. Chan et al., 2013; Dengler et al., 2012; Stefanović et al., 2021). The staphylococcal LTA consists of chains of glycerol‐phosphate (GroP) attached to the plasma membrane through a diglucosyl‐diacylglycerol (Glc_2_DAG) lipid anchor, in a process involving the enzymes UgtP (also referred to as YpfP), LtaA and LtaS (see Schneewind & Missiakas, 2019 for review on LTA biosynthesis). LTAs are believed to not extend through the whole PG mesh and are thus not surface exposed (Matias & Beveridge, 2007; Reichmann et al., 2014). Both WTA and LTA can be decorated with D‐alanyl esters (Reichmann & Gründling, 2011; Xia et al., 2010) and glycosylated with GlcNAc (Rismondo et al., 2021; Winstel et al., 2014) to modulate their charge and properties. In *S. aureus*, it is possible to knock out WTA production, but LTA synthesis cannot be compromised at the same time (Oku et al., 2009). On the other hand, LTA synthase mutants can be obtained, but they usually acquire suppressor mutations and exhibit highly aberrant cell morphologies suggesting that LTA is more important for staphylococcal viability than WTA (Corrigan et al., 2011; Gründling & Schneewind, 2007; Hesser et al., 2020; Oku et al., 2009).

## 4 THE STAGES OF CELL DIVISION

Proper localization of cell wall synthesis and division, is mediated by the essential macromolecular complex, termed the divisome. Bacterial cytokinesis, the physical process of cell division where a parental cell is divided into two identical daughter cells, can be divided into the following three steps: (1) assembly of the Z ring and the divisome, (2) synthesis, constriction, and closure of the division septum, and (3) cell splitting.

### 4.1 Assembly of the Z ring and the divisome

The formation of the division septum is initiated by the polymerization of the tubulin homolog FtsZ into a dynamic filament known as the Z ring (Figure 1c) (Begg & Donachie, 1985; Bi & Lutkenhaus, 1991). The curvature of the FtsZ polymers is referred to as the Z ring, even though a recent study has revealed that FtsZ initially assembles as a D‐shaped structure in staphylococcal cells (Saraiva et al., 2020). The attachment of FtsZ to the inner surface of the cytoplasmic membrane is achieved by FtsA. FtsZ is a highly conserved protein among bacteria, and the Z ring acts as a scaffold for the recruitment of other conserved cell division proteins, which together establish a large complex of proteins called the divisome (Adams & Errington, 2009). The divisome proteins can be divided into two groups according to their temporal pattern of recruitment: (1) the early division proteins, that regulate and stabilize the Z ring, and (2) the late division proteins, including proteins that are critical for the synthesis of the new septal cell wall and constriction of the cytoplasmic membrane (Errington et al., 2003).

FtsZ polymerization represents a key point of control during the bacterial cell cycle (see also Section 5.2). Proteins that have been identified as direct regulators of Z ring formation and part of the early divisome (after FtsZ) include EzrA and SepF. EzrA was first identified in *B. subtilis* as a negative regulator of Z ring assembly (Levin et al., 1999). In *S. aureus*, EzrA interacts with many cell division proteins and is important for cell size homeostasis and for linking late cell wall synthesis proteins (extracellular processes) with the intracellular division ring (Jorge et al., 2011; Steele et al., 2011). SepF was also first identified in *B. subtilis* (Hamoen et al., 2006), but rather as a positive regulator of Z ring formation, promoting bundling of FtsZ protofilaments and suppressing the GTPase activity of FtsZ (Singh et al., 2008). Here, the deletion of *sepF* resulted in septum maturation defects, including abnormally slow septum formation and thick and deformed septa (Hamoen et al., 2006). SepF is conserved in Gram‐positive bacteria. In *S. aureus*, SepF is found to interact with FtsZ and EzrA (Bottomley et al., 2017; Steele et al., 2011), however, the exact function of this protein is still unknown.

The late‐division proteins are dependent on the early FtsZ interacting proteins for localization to the division site (Daniel et al., 2006). GpsB, FtsL, DivIB, and DivIC are regarded as the main late‐division proteins in *S. aureus*, in addition to the PG synthesizing proteins PBP1‐4, RodA, and FtsW (Section 2.1). All the proteins constituting the divisome either have an active role in the synthesis of new PG or in coordinating these processes with cell division (Booth & Lewis, 2019; Pinho et al., 2013). For example, GpsB, a protein conserved within the Firmicutes phylum, has been shown to coordinate PG synthase activity with other cell division processes by interacting with PBPs in *B. subtilis*, *Listeria monocytogenes*, and *S. pneumoniae* (Claessen et al., 2008; Land et al., 2013; Rismondo et al., 2016; Sacco et al., 2022; Tavares et al., 2008). However, in *S. aureus* GpsB appears to modulate and stabilize Z ring assembly and contribute to the remodeling of the divisiome by interacting with FtsZ (Eswara et al., 2018; Sacco et al., 2022). A recent study by Hammond et al. (2022) found that *S. aureus* GpsB directly interacts with TarG, a component of the ABC transporter that exports WTA to the cell surface, thereby coordinating the WTA synthesis machinery with the divisome complex. Indeed, also LTA biosynthesis proteins interact with numerous divisome proteins, strongly suggesting tight coordination of TA synthesis with cell division (Reichmann et al., 2014).

### 4.2 Synthesis, constriction, and closure of the division septum

The Z ring and the divisome do not constitute a static ring structure but move dynamically around as patches in the division plane due to the polymerization and depolymerization of FtsZ (Bisson‐Filho et al., 2017). This dynamic movement, called treadmilling, is particularly important in the early phase of cell division (Monteiro et al., 2018; Whitley et al., 2021). FtsZ treadmilling appears to generate the force needed in the initial slow step of cytokinesis, either by GTP hydrolysis‐induced conformational changes of FtsZ polymers or bundling of FtsZ filaments promoting condensation of the Z ring. During this initial cell cycle phase, PG is incorporated into the lateral cell wall, however, a turning point or checkpoint in cytokinesis occurs when the lipid II flippase MurJ is recruited to the divisome by the late divisome subcomplex DivIB–DivIC–FtsL (Bottomley et al., 2014; Monteiro et al., 2018). MurJ is important for the recruitment of PG synthesis from the cell periphery to the septum due to the lipid II‐affinity of the PG biosynthesis proteins (Monteiro et al., 2018). DivIB and DivIC are cell wall‐binding proteins that also seem to have distinct functions at this checkpoint (Tinajero‐Trejo et al., 2022). Recently it was shown that DivIC has a role in recruiting the PG synthetases PBP2 and FtsW to the division septum, and interestingly, this was shown to depend on the binding of the extracellular domain of DivIC to WTA in the cell wall, suggesting that there may be chemical or architectural characteristics of the cell wall (PG and/or WTA) that is needed to allow further progression of septal synthesis (Tinajero‐Trejo et al., 2022). In addition, it was found that the arrival of MurJ to the septum corresponds to the time point during cytokinesis when the Z ring constriction rate increases (Figure 1d,e). While the initial treadmilling‐dependent phase of cytokinesis is slow, the second step of Z ring constriction appears to be fast and independent of FtsZ treadmilling (Monteiro et al., 2018). PG synthesis and remodeling are believed to be the driving forces of cytokinesis from initial septum formation to cell splitting.

Recent evidence suggests that different PG‐synthesizing enzymes have distinct roles during septal synthesis. As mentioned above, the septum consists of separate layers (an inner core of ordered PG with ring‐like architecture between two layers of mesh‐like PG, Figure 1f) (Pasquina‐Lemonche et al., 2020) which are likely to be synthesized by independent PG synthesis machinery (Straume et al., 2021). Interestingly, it has also been observed that the very first PG in the septum (often termed “piecrust”) is formed independently of PBP1 (Turner et al., 2010; Wacnik et al., 2022). A hypothesis for the division of labor between PG‐synthesizing enzymes is that PBP2 is recruited to the septum immediately after the MurJ‐mediated flipping of lipid II starts and forms the initial PG (Monteiro et al., 2018; Wacnik et al., 2022). Subsequently, the FtsW‐PBP1‐complex synthesizes the ordered PG in the inner septum core, and this layer then works as a framework for the PBP2‐ and PBP4‐mediated synthesis of the mesh‐like outer layers (Wacnik et al., 2022).

The morphology of the septum and localization of PG insertion is also interesting in this context. During synthesis, the septum is thinner at the leading edge, however, when it fuses, a uniform septal thickness is established (Figure 1e,f) (Lund et al., 2018), suggesting that insertion of peptidoglycan does not occur exclusively at the leading edge. Super‐resolution localization microscopy has shown that PG indeed is inserted throughout the septum, and even in the periphery of the cell during the entire cell cycle (Lund et al., 2018).

### 4.3 Daughter cell separation

After new PG is fully synthesized by the divisome, resulting in a septal wall physically separating the two daughter cell compartments, splitting of the mother cell is needed (Figure 1g,h). The actual splitting process happens fast and is over within milliseconds (Monteiro et al., 2015; Zhou et al., 2015). Hydrolases are critical for cell splitting, though, they are not believed to degrade the whole septal wall. Instead, these enzymes initiate the splitting process by hydrolyzing the peptidoglycan “bridge” that connects the daughter cells, and this, together with mechanical factors, results in a sudden crack that separates the cells (Matias & Beveridge, 2007; Zhou et al., 2015).

As mentioned above, *S. aureus* has many different hydrolases (Section 3.2), whose roles are important for both remodeling of PG and cell splitting. The major hydrolase of *S. aureus*, and the best‐characterized one, is Atl. This bi‐functional hydrolase contains two functional domains; an amidase (AmiA), cleaving the linkage between MurNAc and L‐alanine (Biswas et al., 2006), and a glucosaminidase (GlcA) (Oshida et al., 1995). Recent research by Nega et al. (2020) demonstrated that GlcA was dependent on AmiA's activity to the first strip off the stem peptides and that GlcA acted on naked glycan chains only, where it worked as an exoenzyme to release MurNAc‐GlcNAc disaccharides. Two other important hydrolases involved in the cell splitting of *S. aureus* are Sle1 (Kajimura et al., 2005) and LytN (Frankel et al., 2011). Sle1 is an amidase, while LytN functions both, as an amidase and an endopeptidase cutting the D‐Ala‐glycine bond (Frankel et al., 2011; Kajimura et al., 2005).

Cell wall‐splitting hydrolases are known to be regulated transcriptionally by WalK/WalR, as well as other gene regulatory systems (Section 3.2). Importantly, their activity and localization to the septum are also dictated by WTA (Section 3.3). The abundance of mature WTA is probably lower in the septum compared to the old, surrounding cell wall, and mature WTA has been suggested to repel Atl‐derived enzymes, thereby directing this activity to the septum (Schlag et al., 2010). The septal localization of Sle1 and LytN is also dependent on WTA, as WTA‐deficient cells failed to achieve the septal localization of the LysM‐containing cell‐splitting hydrolases (Frankel & Schneewind, 2012). Additionally, Zoll et al. (2012) found that Atl binds to LTA and that it failed to localize at the septal region in an LTA‐deficient strain. Cell‐splitting hydrolases are also regulated at the level of proteolytic degradation; ClpP is a protease that can associate with the chaperone ClpX to create a proteolytic complex (Frees et al., 2003). Jensen et al. (2019) showed that in the absence of ClpX, increased levels of Sle1 resulted in premature cell splitting. ClpX has thus been assigned a regulatory function in controlling the cell splitting of daughter cells.

## 5 COORDINATING STAPHYLOCOCCAL CELL CYCLE PROCESSES

### 5.1 Geometry of cell division

To temporally and spatially coordinate cell division and cell wall synthesis with DNA replication and chromosome segregation, the correct selection of the division plane is crucial (Figure 1). Up until recently, staphylococcal cells were thought to be fully spherical with an intricate geometry of division site selection, in which cell division occurred in three consecutive, perpendicular planes (Koyama et al., 1977; Tzagoloff & Novick, 1977). Recent research has, as mentioned above, shed new light on the details underlying staphylococcal cell morphology and division. Although *S. aureus* appears to have one true PG synthesis machinery, the cells are not fully spherical but elongate slightly during the cell cycle by the action of the RodA/PBP3 complex (Monteiro et al., 2015; Reichmann et al., 2019). After cell splitting, the septal cell wall, therefore, constitutes around one‐third of the new cell wall, in contrast to 50% as previously thought (Monteiro et al., 2018). Notably, Saraiva et al. (2020) also demonstrated that division does not necessarily happen in three consecutive, perpendicular planes, as proposed in a previous model. It was shown that while each division plane is always perpendicular to the previous one, the plane does not have to be perpendicular to the one before that.

### 5.2 Division site selection by a nucleoid occlusion

These new findings (Section 5.1) have important consequences for our understanding of cell division control, that is, when and where the Z ring is formed in the cells (Figure 1). The former theory of division in three alternating perpendicular planes would infer that cells have a form of “memory” of the two previous division planes (Turner et al., 2010), but a mechanism to ensure such memory has never been identified. On the other hand, division in two perpendicular planes does not require memory of the prior division planes, it can rather be explained based on chromosome segregation, nucleoid occlusion, and entropic forces (Saraiva et al., 2020). The nucleoid occlusion protein Noc binds DNA, presumably all over the chromosome but with concentrated levels near *oriC* where it controls DNA replication initiation (Section 2.1) (Pang et al., 2017). Importantly, Noc also inhibits the polymerization of FtsZ and hence the formation of the Z ring. As the origin region is replicated and segregated to the opposite sides of the cell, less Noc will be present at midcell, consequently, the Z ring assembly can start at this Noc‐free location and the division plane is thereby defined (Veiga et al., 2011). When the septum is formed, the cell consists of two temporarily asymmetrical daughter cell compartments with different longitudinal axes. Due to spatial constraints and entropy, chromosome segregation will occur along the longer axis, which is parallel to the division septum. After chromosome segregation, only one possible division plane, that does not bisect the nucleoid containing Noc, will be available, and this plane is inevitably perpendicular to the previous one (Jun & Wright, 2010; Saraiva et al., 2020).

### 5.3 CcrZ is a novel cell cycle control protein

**Figure 3:**
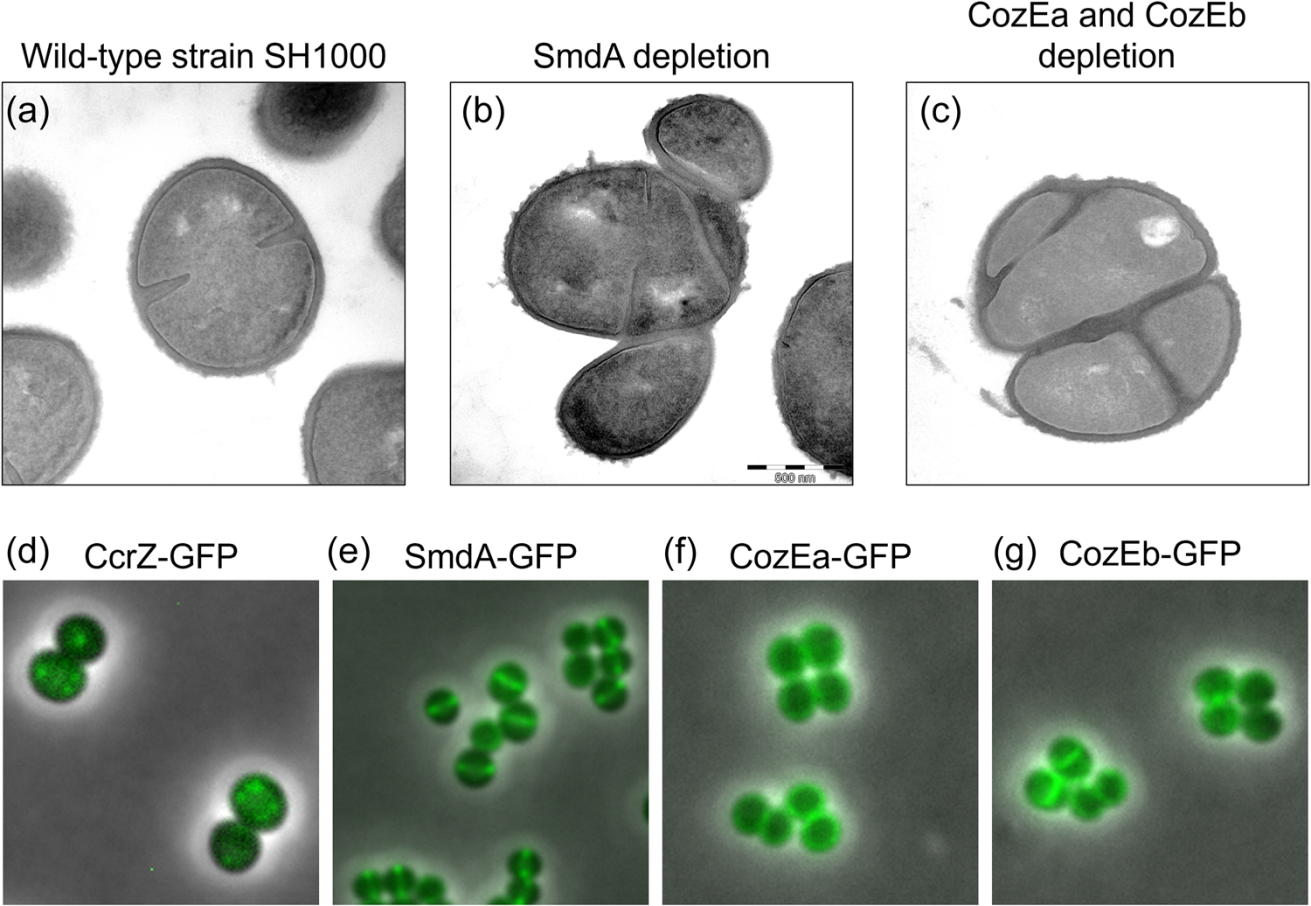
Phenotypes and localization of novel cell cycle factors. Transmission electron micrographs showing typical phenotypes of *Staphylococcus aureus* SH1000 wild‐type (a), cells depleted of SmdA (b), and cells depleted of CozEa and CozEb (c). Merged phase contrast and fluorescence images showing the subcellular localization of CcrZ (in cytoplasmic foci, d), SmdA (septum‐enriched, e), CozEa (membrane‐localization, f), and CozEb (membrane‐localization, g).

Although less characterized, other mechanisms likely also play a role in coordinating Z ring formation with other cell cycle processes in *S. aureus*. One of these mechanisms likely involves the DNA replication initiation regulator CcrZ (Section 2.1). Gallay et al. (2021) observed that CcrZ in ovococcal *S. pneumoniae* localizes to new cell division sites through direct interactions with FtsZ, where it stimulates DnaA to initiate DNA replication. The absence of CcrZ caused several division defects, including anucleate cells, cleaved chromosomes, multiple and aberrant division septa, and dramatic growth rate reduction, as a result of mistimed and reduced initiation of DNA replication. The functions of CcrZ appear to be conserved in *S. aureus* and *B. subtilis*, and probably other Gram‐positive bacteria since similar phenotypes are observed, although it should be noted that CcrZ localizes in foci and not along the Z ring in these species (Figure 3).

## 6 OTHER FACTORS AFFECTING THE CELL CYCLE PROGRESSION AND CELL MORPHOLOGY

A number of control mechanisms to keep the cell division proteins correctly localized have been described in the previous sections. These include localization and activity control via dynamic protein–protein or protein–cell wall interactions, transcriptional regulation, and proteolytic degradation. General processes such as the secretion and translocation of proteins and molecules across the membrane also play important roles. For example, since molecules need to move across the membrane to execute their function, the secretion‐associated proteins SecDF are important for normal cell division and cell morphology in *S. aureus* (Quiblier et al., 2011). Moreover, protein phosphorylation modulates the activity of key cell division and cell wall synthesis proteins in a diversity of bacterial species. In addition to CcrZ discussed above, the serine/threonine protein kinases are of particular importance (Grangeasse, 2016). These proteins harbor extracellular PG binding PASTA domains and an intracellular kinase domain, allowing intracellular phosphorylation of proteins in response to changes in the extracellular cell wall. In *S. aureus*, the serine/threonine protein kinase PknB (also known as Stk1 or Stk) and the cognate phosphatase Stp have been shown to modulate the cell cycle at different levels, possibly in response to the levels of the cell wall precursor lipid II (Hardt et al., 2017; Jarick et al., 2018).

In addition to the factors mentioned above, a number of proteins for which the mechanisms are not yet fully characterized, have recently been found to have major effects on staphylococcal cell morphology and division. An example of this is CozE (coordinator of zonal elongation), a family of multitransmembrane proteins that are broadly distributed across the bacterial kingdom (Fenton et al., 2016). The CozE proteins were first identified in *S. pneumoniae* as an important contributor to cell elongation and morphology, potentially by controlling activities of PBPs, particularly the bifunctional PBP1a (Fenton et al., 2016; Stamsås et al., 2020). *S. aureus* encodes two CozE paralogs, which seem to have overlapping functions (Stamsås et al., 2018), as single deletions were viable while a double knockout was lethal (Figure 3). Double *cozE* knockdown had major effects on the morphology of *S. aureus*, as demonstrated by cells displaying both thicker septa and problems with the initiation of septum formation, and disturbances in the chromosome biology observed by abnormal staining patterns of the nucleoids, suggesting that these proteins control proper cell cycle progression in *S. aureus* (Stamsås et al., 2018).

Another example is SmdA (staphylococcal morphology determinant A), which is a *Staphylococcus*‐specific factor shown to affect cell morphology. This membrane‐attached, septum‐enriched protein interacts with proteins in the divisome and is critical for maintaining proper cell morphology in *S. aureus* (Figure 3) (Myrbråten et al., 2022). Knockdown of SmdA resulted in cell division defects, including increased cell clustering of misshaped cells, misplaced septum synthesis, and abnormal nucleoid staining (Myrbråten et al., 2022). Notably, SmdA interacts with PBPs and EzrA, and the knockdown of SmdA results in increased sensitivity to cell wall targeting antibiotics, including resensitization to β‐lactams, which is a feature shared among several cell division proteins (Bilyk et al., 2022; Myrbråten et al., 2022).

A final example of a newly identified *Staphylococcus* cell cycle factor is SosA, a cell division inhibitor that is induced by the SOS response in the event of DNA damage to ensure that the DNA is repaired before cell division is continued (Bojer et al., 2019). Microscopy and localization data suggest that SosA accumulation inhibits cell division without causing the divisome to delocalize and without affecting the placement of EzrA or GpsB. In contrast to related proteins in other bacteria, SosA in *S. aureus* lacks a PG‐binding LysM‐domain, and exactly how SosA affects divisome activity is unclear, although it appears to happen sometime between septum initiation and septum completion (Bojer et al., 2019).

## 7 SUMMARY

Recent progress in genetics and cell biology, in particular when it comes to localization microscopy techniques, has accelerated the research and our understanding of cell cycle processes in a range of bacterial species. However, even for the well‐studied species, such as *S. aureus*, we have only begun to fully reveal the intricate spatial and temporal relationships between the different cell cycle processes. Indeed, novel cell cycle factors are continuously being identified, and obtaining a complete and integrated mechanistic understanding is still a major challenge for future research. It is of particular interest to understand the interplay between cell division and chromosome biology in coccus‐shaped *S. aureus* since the mechanisms clearly differ from other model bacteria with elongated cell shapes.

## AUTHOR CONTRIBUTIONS

**Maria D. Barbuti**: Conceptualization (equal); writing – original draft (lead); writing – review & editing (equal). **Ine S. Myrbråten**: Conceptualization (equal); writing – original draft (equal). **Danae Morales Angeles**: Conceptualization (supporting); writing – review & editing (supporting). **Morten Kjos**: Conceptualization (equal); writing – original draft (supporting); writing – review & editing (lead).

## CONFLICT OF INTEREST

None declared.

## ETHICS STATEMENT

None required.

## Data Availability

Not applicable.
